# Improvements in Glucose Sensitivity and Stability of *Trichoderma reesei* β-Glucosidase Using Site-Directed Mutagenesis

**DOI:** 10.1371/journal.pone.0147301

**Published:** 2016-01-20

**Authors:** Boyang Guo, Yoshihiko Amano, Kouichi Nozaki

**Affiliations:** Department of Bioscience and Textile Technology, Interdisciplinary Graduate School of Science and Technology, Shinshu University, Nagano, Japan; Weizmann Institute of Science, ISRAEL

## Abstract

Glucose sensitivity and pH and thermal stabilities of *Trichoderma reesei* Cel1A (Bgl II) were improved by site-directed mutagenesis of only two amino acid residues (L167W or P172L) at the entrance of the active site. The Cel1A mutant showed high glucose tolerance (50% of inhibitory concentration = 650 mM), glucose stimulation (2.0 fold at 50 mM glucose), and enhanced specific activity (2.4-fold) compared with those of the wild-type Cel1A. Furthermore, the mutant enzyme showed stability at a wide pH range of 4.5–9.0 and possessed high thermal stability up to 50°C with 80% of the residual activities compared with the stability seen at the pH range of 6.5–7.0 and temperatures of up to 40°C in the wild-type Cel1A. Kinetic studies for hydrolysis revealed that the Cel1A mutant was competitively inhibited by glucose at similar levels as the wild-type enzyme. Additionally, the mutant enzyme exhibited substrate inhibition, which gradually disappeared with an increasing glucose concentration. These data suggest that the glucose stimulation was caused by relieve the substrate inhibition in the presence of glucose. To conclude, all the properties improved by the mutagenesis would be great advantages in degradation of cellulosic biomass together with cellulases.

## Introduction

β-Glucosidases (BGLs) are widely distributed in nature for the liberation of glucose from cello-oligosaccharides and glucosides linked by a β-1,4-glycosidic bond. In particular, fungal and bacterial BGLs are responsible for the final saccharification step in producing fermentable sugar and in preventing the product inhibition of exo- and endoglucanases during the degradation of cellulosic biomass [1–7]. However, most BGLs are commonly inhibited by glucose [8]. This is caused by the competitive binding of substrate and glucose to the same active site on the enzyme. Cel1A (Bgl II) from *Trichoderma reesei*, an industrial cellulase producer, although the location of Cel1A was still unclear by so far, however some evidences indicated the major BGL activity from *Trichoderma reesei* was detected in extraction of plasma-membrane, thus Cel1A is probably as a cell-membrane bonding enzyme. Moreover some other research evidenced Cel1A catalysis transglycosylation reactions product sophorose, laminaribiose and gentiobiose from an extremely high concentration of cellobiose (10%) [9]. It is also reported inhibited by glucose with *K*_i_ approaching 50 mM during the hydrolysis of the substrate *p*-nitrophenyl β-D-glucoside (pNPG) [10,11]. In contrast, several glucose tolerant or -stimulated BGLs belonging to the glycoside hydrolase family (GH) 1 or 3 have been isolated and characterized [12–23]. *Humicola insolens* BGL (HiBGL) belongs to GH1, specifically shows significant homology to the amino acid sequence of Cel1A with 73% identity and 82% similarity. This enzyme exhibits high glucose tolerance and enhances 1.8 fold activity at 50 mM glucose or 100 mM xylose [11,16,24]. HiBGL is also stable at the pH range of 5.0–8.0 and shows high thermostability with a half-life of 43.8 min at 55°C [17]. Furthermore, the amino acid residues of Cel1A and HiBGL, which comprise subsites −1 and +1 as well as two catalytic residues, were completely conserved. Focusing on the active site entrance, seven amino acid residues are different from each other. Some of these amino acids in Cel1A were substituted with different kinds of amino acid residues and their subsequent properties were investigated [25]. Most mutations influenced kinetic parameters and/or thermostability; e.g., P172L and P172L/F250A mutants enhanced the catalytic efficiency (*V*_max_/*K*_m_), while L167W and P172L/F250A mutants increased the optimum temperature to 50°C [25]. Further, the single mutants (L167W or P172L) markedly increased glucose tolerance [25]. However, higher thermal stability and glucose stimulation evident in HiBGL was not observed in these mutants [25]. Among the seven different amino acids in HiBGL, W168, L173, and F348 might be responsible for glucose stimulation and tolerance, restricted the volume and width of the active site entrance and trapped a glucose molecule in subsite +2 through hydrophobic interactions [11,24]. In this study, we performed site-directed mutagenesis for the three corresponding amino acid residues in Cel1A, L167, P172 and P338 to improve glucose sensitivity and pH and thermal stabilities.

## Materials and Methods

### Cloning and Site-directed Mutagenesis

*T*. *reesei* QM9414 strain (ATCC 26921) was used for nucleic acid isolation. *E*. *coli* DH5α was used for the routine propagation of all plasmids and *E*. *coli* Rosetta-Gami 2 (DE3) pLysS were used as a host strain for protein expression, all of these strains are purchased by Merck, Darmstadt, Germany. RNA was isolated using TRIzol reagent (Invitrogen, Carlsbad, CA) according to the manufacturer’s instructions. Synthesis of cDNA was carried out using a PrimeScript^™^ II 1st strand cDNA Synthesis Kit (TaKaRa Bio Inc., Shiga, Japan) according to the manufacturer’s instructions. The synthesized cDNA was further used as template for BGL encoding cDNA amplification by PCR with PrimeSTAR^®^ HS DNA polymerase (TaKaRa Bio Inc). Primer pairs (Cel1A-F and Cel1A-R) containing additional NcoI and HindIII sites are listed in Table 1. The amplified cDNA was inserted into the same sites of pET23d (+) vector (Merck, Darmstadt, Germany) to encode a Histidine-tag fusion at the C-terminus. Mutagenesis was performed by overlapped PCR using PrimeSTAR^®^ Max DNA polymerase (TaKaRa) with the aforementioned resultant plasmid. Forward and reverse primers are also listed in Table 1, in which the nucleotide sequences partially overlapped. Expression vectors for double and triple mutations were also constructed by repetitive mutagenesis in the same way. The resulting amplicon was introduced into *Escherichia coli* DH5α, and the mutated plasmid was then prepared using QIAprep Spin Miniprep Kit (QIAGEN, Hilden, Germany). All nucleotide sequences of the expression plasmids were confirmed by sequencing.

**Table 1:** List of PCR primers.

Primer name	Sequence (5’–3’)
Cel1A-F	AccatggTGCCCAAGGACTTTCAGTGG
Cel1A-R	CAaagcttCGCCGCAATCAGCTCGT
L167W-F	ACGAGCCGTGGTGCTCGGCCATCC
L167W-R	AGCACCACGGCTCGTTGAAGGTGA
P172L-F	CATCCTGGGCTACGGCTCCGGCAC
P172L-R	CCGTAGCCCAGGATGGCCGAGCAC
P338F-F	CGCAGTCCTTCTGGCTGCGCCCCT
P338F-R	GCCAGAAGGACTGCGTCTCGGGGC

The introduced restriction sites are indicated as small letters. The overlapped sequence is underlined.

### Expression and Purification

Each plasmid for expression was introduced into the *E*. *coli* Origami B (DE3) pLysS strain. The resultant single colony was grown in 100 ml of LB liquid medium (Nakalai Tesque, Kyoto, Japan) supplemented with 100 μg/ml of ampicillin in a 1-l baffled flask on a rotary shaker (160 rpm) at 25°C. Furthermore, 1 mM of isopropyl β-D-thiogalactopyranoside was added as an inducer when the absorbance reached 0.6 at 660 nm. After the next 12 h of growth, the cells were harvested by centrifugation (13,000 ×*g* for 5 min) and were then re-suspended in 15 ml of 20 mM phosphate buffer (pH 7.5) containing 0.1 M NaCl. After ultrasonication for 10 min on ice, cell debris was removed by centrifugation (13,000 ×*g* for 5 min). Protein purification was then conducted with the supernatant using a column packed with 15 ml of the TALON metal affinity resin (TaKaRa) equilibrated with the same buffer. The proteins adsorbed to the column were eluted with 200 mM imidazole in the same buffer. The imidazole in the pooled enzyme solution was removed, and the buffer was substituted with 20 mM acetate buffer (pH 5.5) by gel filtration using a PD-10 column (GE Healthcare, WI, USA). The purities of the recombinant BGLs were confirmed by sodium dodecyl sulfate-polyacrylamide gel electrophoresis (S1 Fig).

### Enzyme Assay

BGL activity was assayed in a 0.4 ml of reaction mixture containing 1.25 mM *p*-nitrophenyl β-D-glucoside (pNPG), 100 mM acetate buffer (pH 5.5), and a suitable amount of enzyme. After incubation for 0–30 min at 30°C, 2 ml of 0.5% Na_2_CO_3_ was added. The absorbance of the released *p*-nitrophenol was determined at 420 nm. Activity units (U) were calculated from an initial velocity, and 1 U was defined as the amount of enzyme releasing 1 μmol of *p*-nitrophenol per min. Protein content was measured by the Lowry method [26] using bovine serum albumin as the standard. The transglycosylation ability was also measured under the same conditions as described in the BGL activity using various concentrations of substrate and 50 μg of proteins. After reaction, the products were detected by the thin layer chromatography.

Parameters of glucose inhibition for hydrolysis (*K*_i_) were calculated from Dixon plots [27] using initial velocities. The plots were specially used for calculating in the case of low substrate concentration (0.060–0.34 mM in the absence of glucose and 0.060–2.1 mM for 10 mM glucose addition). Kinetic parameters for hydrolysis were also determined using the Lineweaver–Burk plots after measuring initial reaction velocities at various substrate concentrations (0.060–2.5 mM) [28].

### Effects of pH and Temperature on Activity and Stability

Optimum pH and temperature were measured under the conditions described in the enzyme assay except where the buffer was substituted with Britton–Robinson’s wide range buffer and the reaction temperatures were shifted to 30–65°C, respectively. Stability for pH and temperature were also determined under the same assay conditions after the enzyme was incubated at various pH for 6 h at 4°C and at various temperatures for 30 min at pH 5.5, respectively.

## Results and Discussion

### Structural Analysis of Cel1A and HiBGL

**Fig 1:**
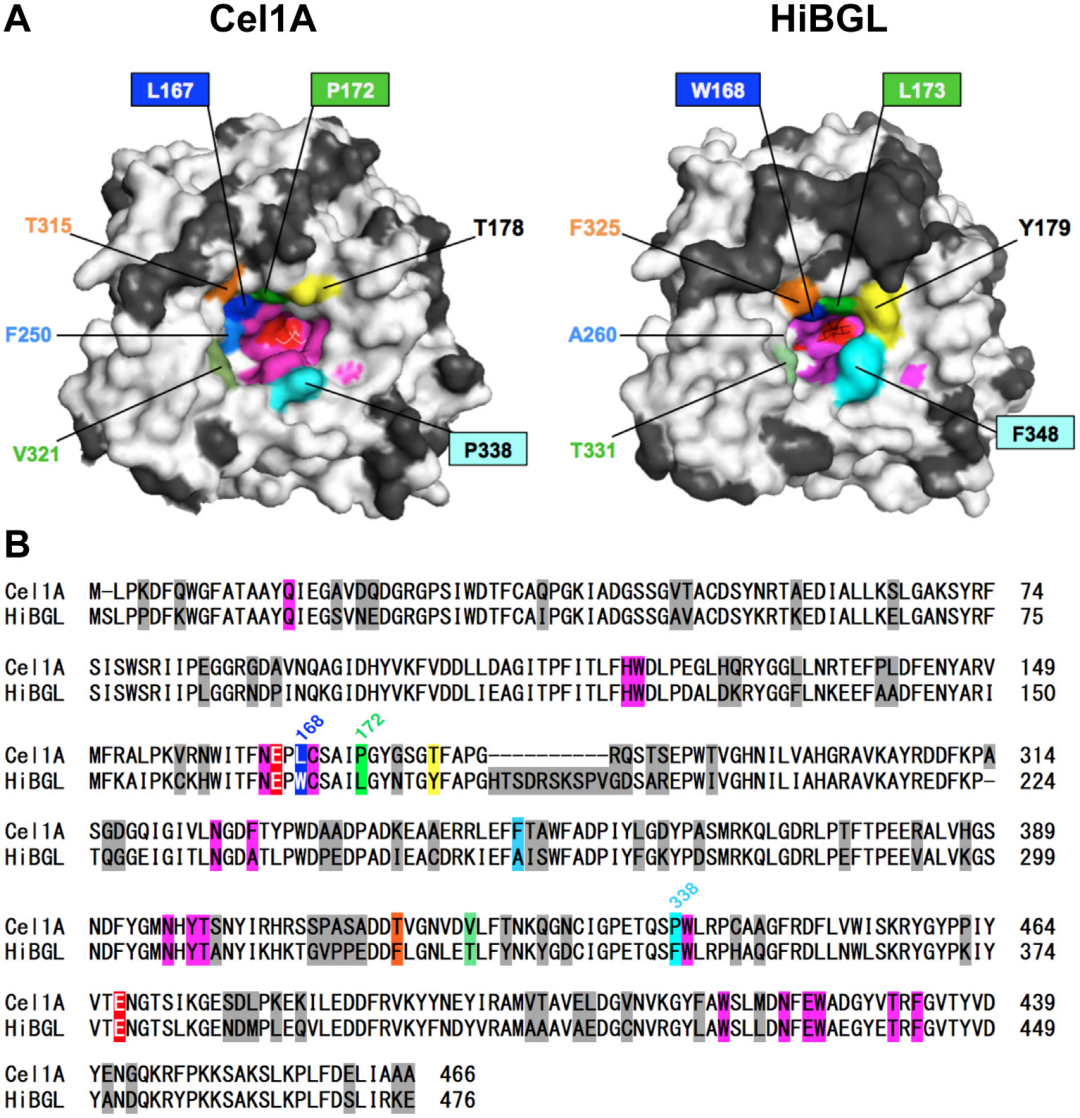
Comparison of the structures between Cel1A (PDB entry 3ahy) and HiBGL (PDB entry 4mdp). **(A)** 3D structure on the side of the active site entrance. white, identical or similar amino acids; black, different amino acids; red, catalytic residues; pink, subsite −1 and +1; other colors, different amino acid residues located at the active site entrance. **(B)** Alignment of amino acid residues of Cel1A and HiBGL.

We compared the crystal structures of Cel1A [10] and HiBGL [11] to plan the experimental strategy for mutagenesis (Fig 1A and 1B). Around the active site entrance, seven amino acid residues different to each other can be observed. These are important in shaping the entrance and cavity along the active site tunnel. Three of these amino acids (W168, L173, and F348) in HiBGL were also reported to correspond with subsite +2 and trap the glucose molecule by hydrophobic interaction. Additionally, W168 and L173 of HiBGL were found to restrict the width of the active site and maintain a narrow cavity [11]. In this study, we tried to substitute the corresponding amino acids L167, P172, and P338 in Cel1A by creating L167W/P172L and L167W/P172L/P338F mutants of Cel1A. They were named as 167/172 and 167/172/338 mutants, respectively.

### Glucose Tolerance and Stimulation of Cel1A Mutants

**Fig 2:**
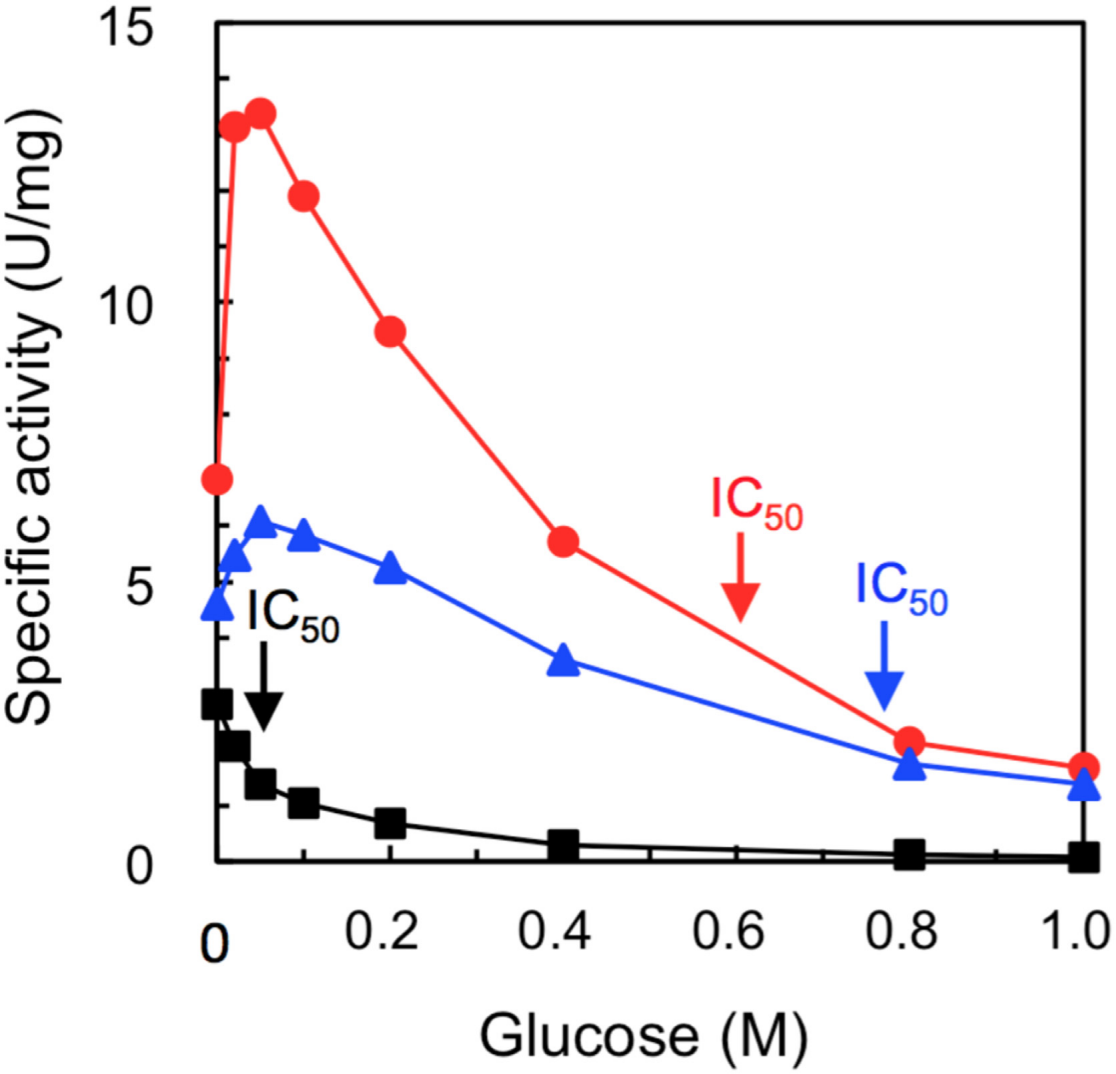
Effect of glucose on BGL activity. Hydrolytic activities at 1.25 mM of pNPG were determined in the presence of glucose. ■, WT; ●, the 167/172 mutant; ▲, the 167/172/338 mutant. Plots are averages of independent triplicates. The arrowed IC_50_ values were calculated by the concentration of inhibitor that halves the enzyme activity without inhibitor.

The mutants of amino acids 167/172 and 167/172/338 had significantly increased specific activities (6.8 U/mg for 167/172 and 4.6 U/mg for 167/172/338) compared with those of wild-type Cel1A (WT, 2.8 U/mg) (Fig 2 and Table 2). Furthermore, we show for the first time that the activities of the mutant enzymes continued to be stimulated with an increasing glucose concentration (Fig 2). The maximum stimulation activity reached up to 2.0 (the 167/172 mutant) and 1.3 fold (the 167/172/338 mutant) at 50 mM glucose. The stimulatory behavior of the 167/172 mutant is similar to that of HiBGL (1.8 fold stimulation at 50 mM glucose) [24]. This indicates that both L167W and P172L mutations are indispensable for glucose stimulation because each single mutant was reported to show no stimulation of glucose [25]. Though it is necessary for HiBGL to interact with glucose, the additional P338F mutation appeared to perturb glucose stimulation this might be explained by the arrangement and orientation of P338 in Cel1A, which would be different from that of F348 in HiBGL, as the P338 residue was located at a considerable distance from the L167 and P172 residues.

**Table 2:** Kinetic parameters of WT and the 167/172 mutant for hydrolysis of pNPG.

Parameters and property	WT	167/172 mutant
Specific activity (U/mg) *	2.8	6.8
*V*_max_ (U/mg)	7.7 ± 1.62	13.3 ± 0.34
*K*_m_ (mM)	2.48 ± 0.75	0.23 ± 0.01
*V*_max_/*K*_m_	3.06	57.7
*K*_i_ for glucose (mM)	50	50
IC_50_ (mM)	600	50
Substrate inhibition	−	+

* determined under the same conditions as the enzyme assay.

In contrast, the activity of the two mutants gradually decreased above a glucose concentration of 50 mM. However, both mutants showed glucose tolerance with the same 50% of inhibitory concentration (IC_50_) of 600 mM, which was markedly higher than the IC_50_ value of 50 mM of WT (Fig 2). These tolerances approximately corresponded to the IC_50_ value of 800 mM of HiBGL at 0.2 mM pNPG as the substrate [24]. The same tolerance was also reported previously in the L167W or P172L single mutant [25]. From these data, we concluded that either L167W or P172L, but not both, are necessary for glucose tolerance. All the aforementioned data suggest that the mechanisms of glucose stimulation and tolerance are distinguished. A previous report suggested that the glucose tolerance is caused by the narrower cavity of the active site preventing the incorporation of glucose into the active site on the enzyme [11].

Glucose-tolerant BGLs often accompany glucose stimulation [13,15–23,29,30]. Among these BGLs, Td2F2 enhances the apparent hydrolytic activity to pNPG with an increase in glucose concentration by transglycosylation, leading to the formation of disaccharides such as sophorose [20]. For an understanding on whether the glucose stimulation and tolerance is concerned with the transglycosylation, we thus carried out. However, Cel1A and both mutants in this study accumulated no transfer products. The transglycosylation products were generally considered to accumulate at higher concentration of donor and acceptor. We concluded that these mutants enhance hydrolytic activity in the presence of glucose.

### pH and Temperature Effects on Activity and Stability

**Fig 3:**
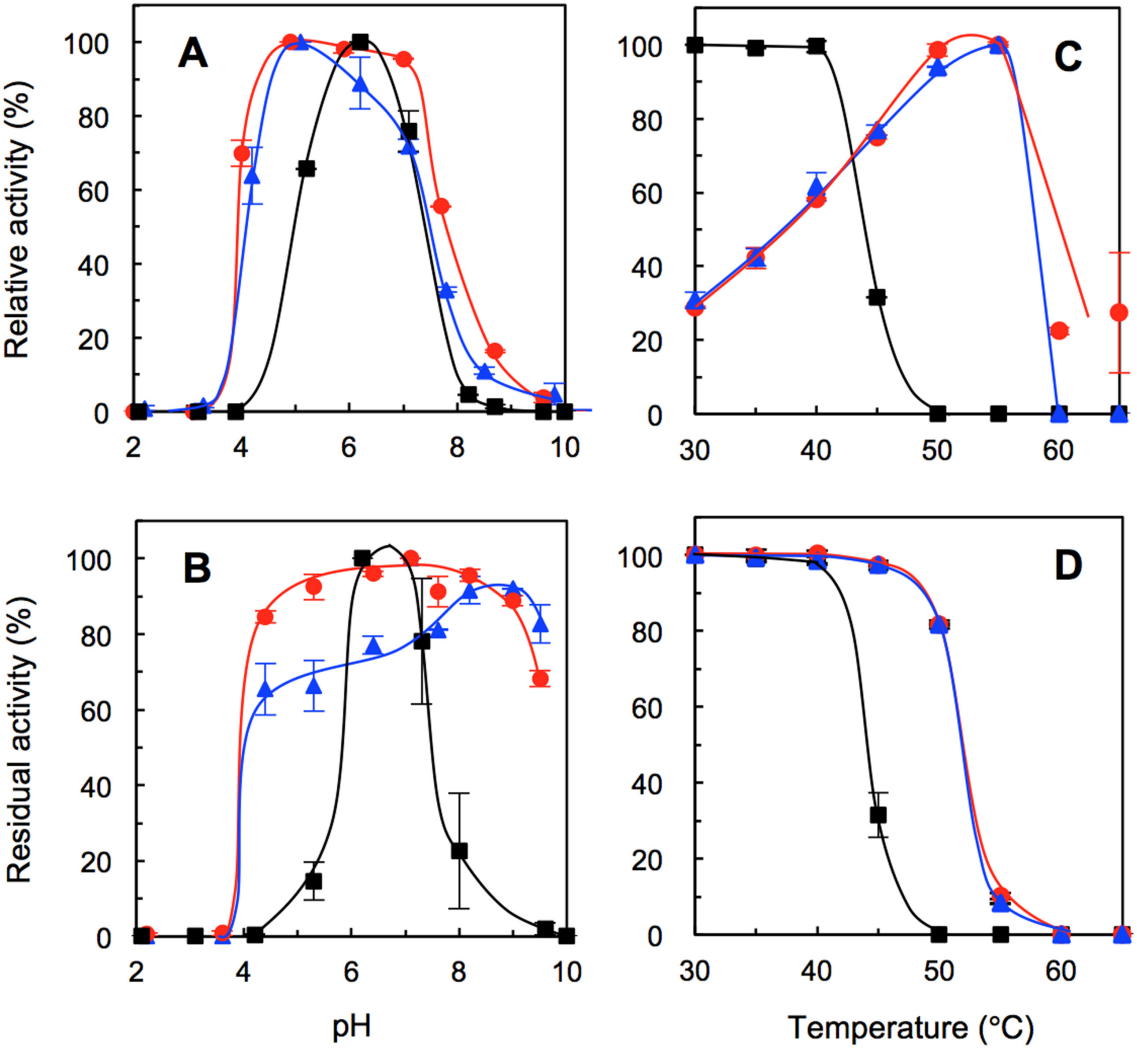
pH and Temperature profiles of WT and the 167/172 and the 167/172/338 mutants. **(A)** Optimum pH, **(B)** pH stability, **(C)** Optimum temperature, **(D)** Thermal stability. ■, WT; ●, the 167/172 mutant; ▲, the 167/172/338 mutant. Vertical bars indicate the standard deviations of independent triplicates.

The optimum pH of the two mutant BGLs expanded in the acidic region and exhibited high activity in the pH range of 5.0–7.0 (Fig 3A), which was different from the pH range of 6.0–6.5 of HiBGL [17]. Stabilities for pH also markedly increased, showing 80% of the residual activities at a wider pH range of 4.0–9.0 (the 167/172 mutant) and 7.5–9.5 (the 167/172/338 mutant) (Fig 3B). The profile of pH stability of the 167/172/338 mutant closely resembles that of HiBGL [17]. The 167/172 mutant kept activity at the pH range of 4.0–7.0 and was higher than the activity of the 167/172/338 mutant and HiBGL [17].

The optimum temperatures of both mutants shifted to 55°C, which was higher than that of 30–40°C of WT (Fig 3C) but slightly lower than that of HiBGL (60°C) [17]. The thermal stabilities of the two mutants were also equally increased to 50°C compared with that of WT, which was 40°C (>80% of the residual activity) (Fig 3D). These data corroborated the *T*_m_ values of the 167/172 and the 167/172/338 mutants, which increased to 59 and 57°C, respectively, compared with that of WT (52°C) (S2 Fig). Lee et al. reported that the Cel1A mutant of L167W or P172L increased the *T*_m_ values to 54.9 and 54°C, respectively [25]. This suggests that both the L167W and P172L mutations synergistically increased thermostability but that the additional P338F mutation was not necessary. However, we were unable to clarify the mechanism for increased thermostability.

### Kinetic Studies

**Fig 4:**
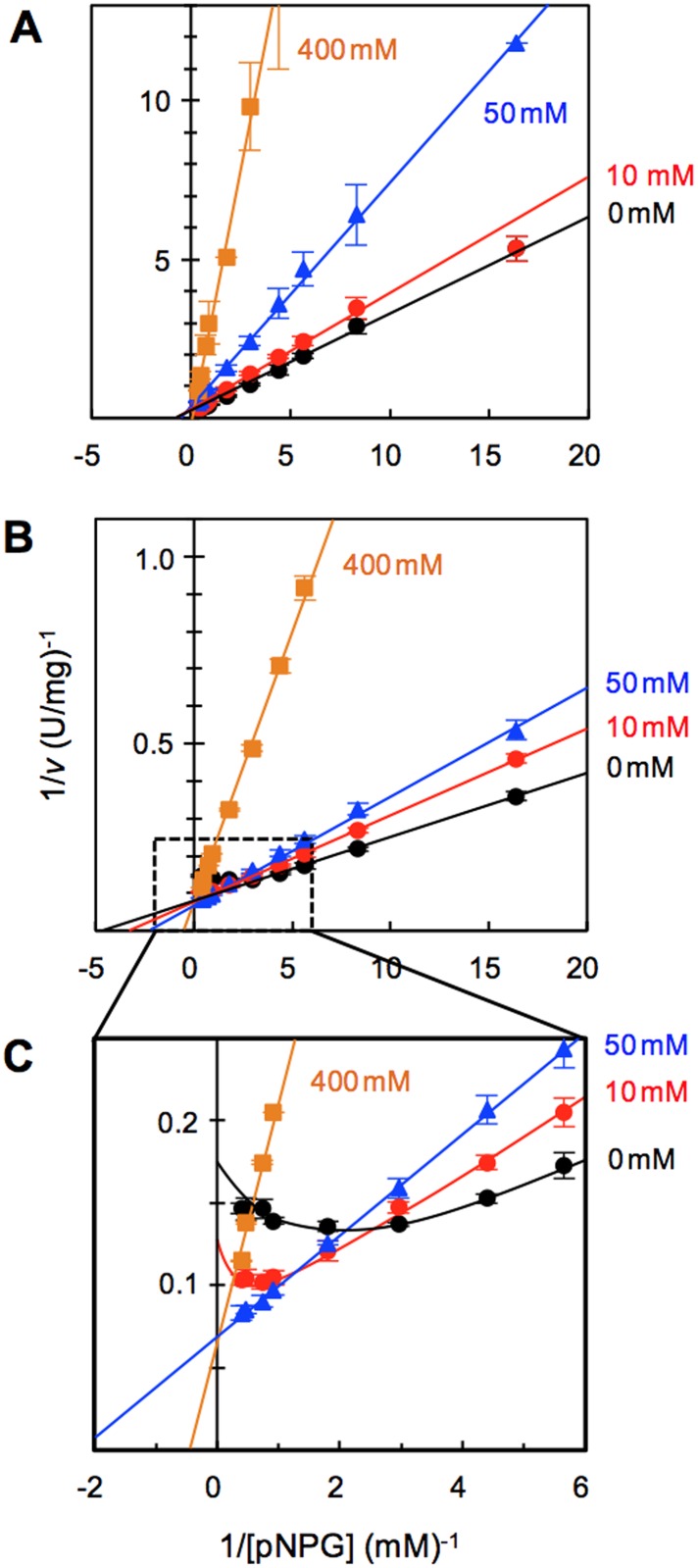
Kinetic analysis of WT and the 167/172 mutant in the presence of glucose. **(A)** WT, **(B)** The 167/172 mutant, **(C)** Enlarged view of Fig 3B at high concentration of substrate. Glucose concentrations were shown in figure. Vertical bars indicate the standard deviations of independent triplicates.

To elucidate the catalytic properties, kinetic analysis was performed on the 167/172 mutant using pNPG as the substrate (Fig 4 and S3 Fig). *K*_m_ of the 167/172 mutant (0.23 mM) was much smaller than that of WT, which was 2.48 mM, and similar to that of HiBGL, which was 0.16 mM [17]. *V*_max_ of the 167/172 mutant, which was 13.3 U/mg, was higher than that of WT, which was 7.7 U/mg, but was slightly lower than that of HiBGL, which was 18.7 U/mg (Table 2). In conclusion, the catalytic efficiency (*V*_max_/*K*_m_) of the 167/172 mutant was 19 fold higher than that of WT. The enhanced catalytic efficiency was mainly due to the markedly decreased *K*_m_. All these data were similar to the results obtained from the single mutation of L167W or P172L in Cel1A, but the double mutant in this study enhanced the effect [25].

The mutant showed competitive inhibition in the presence of glucose at low concentrations of substrate (<0.25 mM), which was similar to that of WT (Fig 4A and 4B). The Dixon plots analysis also revealed that the inhibitory parameter (*K*_i_) of the 167/172 mutant was nearly identical to 50 mM of WT (S4 Fig). The 167/172 mutant showed substrate inhibition, which was not observed in WT, at higher concentrations of pNPG (>0.25 mM) in the absence of glucose (Fig 4C and S3 Fig). When glucose concentration was increased, substrate inhibition was gradually prevented. At 50 mM glucose (the maximum concentration for stimulation), the substrate inhibition completely disappeared. Consequently, the hydrolytic activity was stimulated because the hydrolytic velocity faithfully adhered to the model of Michaelis–Menten kinetics [31] without substrate inhibition (Fig 4C and S3 Fig). The same figure also shows that glucose acts as both an activator and inhibitor. Above the concentration of 0.25 mM of pNPG in the presence of 0–50 mM glucose, it would be difficult to determine accurate kinetic and inhibitory parameters due to the co-existence of glucose stimulation, substrate inhibition, and glucose inhibition.

**Fig 5:**
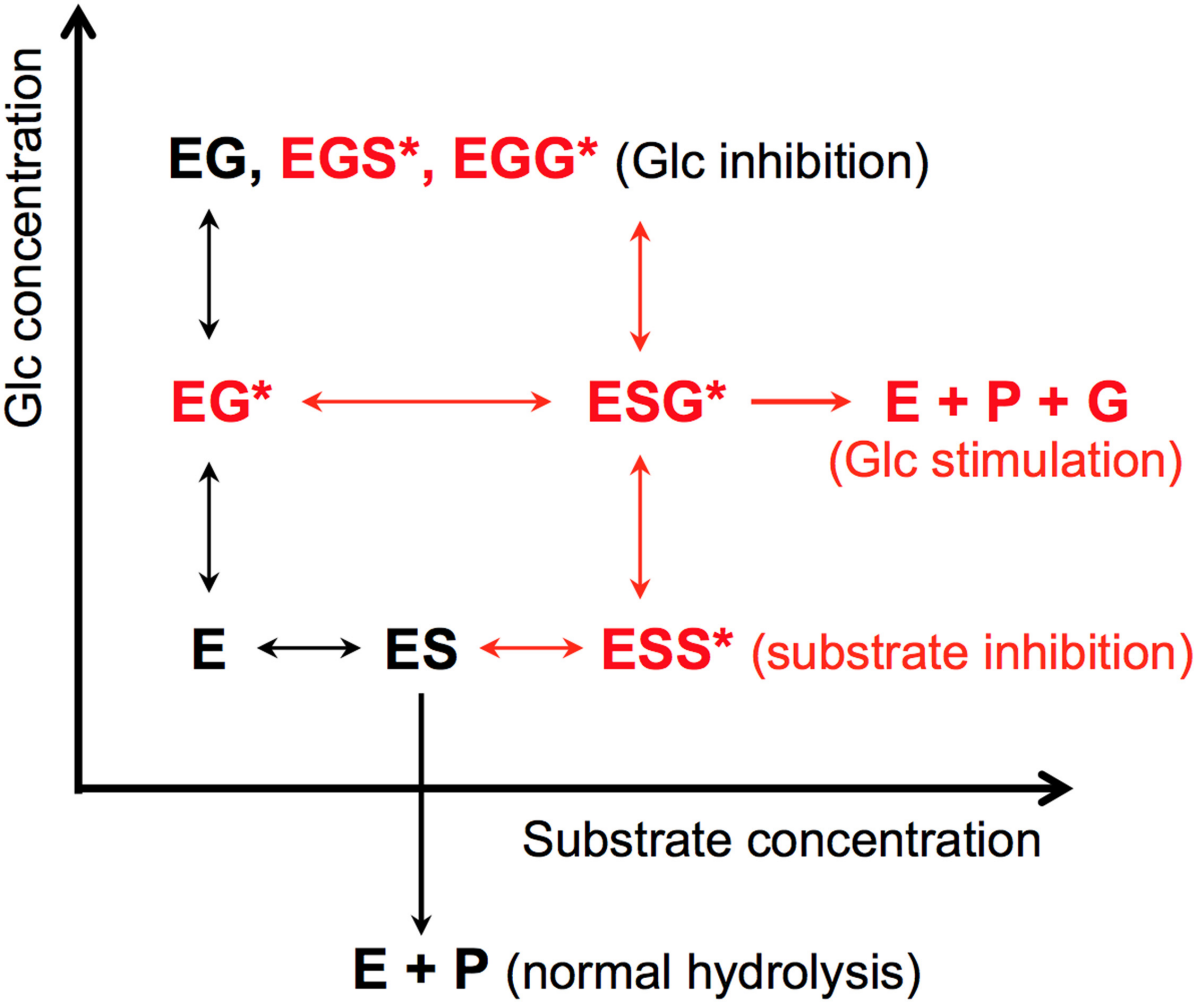
Schematic representation of the reaction sequences of WT and the 167/172 mutant. E, enzyme; S, substrate; G, glucose. The reactions shown in red occur only in the 167/172 mutant. Asterisks indicate the binding to the introduced secondary site in the 167/172 mutant.

Souza et al. suggested that glucose stimulation in HiBGL is induced by the conformational change of the enzyme binding to glucose at the modular-binding site [24]. Our data do not disprove their estimations, but we have further clarified the mechanism of glucose stimulation (Fig 5). Substrate inhibition is induced by another substrate molecule binding to a secondary site, the non-active site, on the enzyme. The introduced mutation (L167W/P172L) might create a secondary substrate-binding site. The binding of the substrate to this site inhibits the hydrolysis forming ESS* complex (* indicate the binding to the created secondary site). When glucose is present (<50 mM), the substrate at the secondary site might be replaced by glucose forming ESG* complex as reported in case of HiBGL [11] and glucose stimulation was caused by relieve the substrate inhibition. When glucose concentrations are elevated to over 50 mM, most of the subsites (-1 and +1) and the secondary site are occupied by glucose leading to EG, EGS* or EGG* complexes. As a result, a competitive glucose inhibition was caused as shown in WT.

## Conclusions

The mutagenesis of only two amino acid residues (167/172) in Cel1A improved glucose tolerance, glucose stimulation, and pH and thermal stabilities, while also markedly increasing the specific activity. All these properties would be a great advantage in the degradation of cellulose together with cellulases. In addition to this, Cel1A is an important enzyme, which converts cellobiose to sophorose, a powerful inducer of cellulase from *T*. *reesei* [32,33]. We believe that the mutant enzyme introduced into *T*. *reesei* enhances both cellulase induction and production.

## Supporting Information

S1 Fig SDS-PAGE analysis of the purified WT and the 167/172 and the 167/172/338 mutants. The recombinant enzymes (5 μg each) were subjected to electrophoresis. M, molecular mass markers.
(PDF)

S2 Fig Melting temperatures of WT and the 167/172 mutant. Melting temperature was determined using Protein Thermal Shift Dye Kit (Thermo Fischer Scientific Inc., MA, USA) according to the instruction manual. A melting curve was created to trace the fluorescence (Ex/Em = 580 nm/610 nm) from 40 to 70°C at an increasing rate of 0.05°C per second. The melting temperature (*T*_m_) was then obtained from a differential curve calculated from the melting curve.
(PDF)

S3 Fig Velocity vs [S] plots of WT and the 167/172 mutant in the presence of glucose. **(A)** WT, **(B)** The 167/172 mutant. The same data from Fig 4 were plotted. Glucose concentrations were shown in figure. Vertical bars indicate the standard deviations of independent triplicates.
(PDF)

S4 Fig Dixon plot analysis for determination of parameters in glucose inhibition. **(A)** WT, **(B)** The 167/172 mutant. The same data from Fig 4 were plotted. Plots are averages of independent triplicates.
(PDF)

## References

[1] Baba Y, Sumitani J, Tani S, Kawaguchi T (2015) Characterization of β-glucosidase 1 accelerating cellulose hydrolysis with cellulase system. AMB Express 5: 3. 25642400 10.1186/s13568-014-0090-3 PMC4305095

[2] Wang B, Xia L (2011) High efficient expression of cellobiase gene from *Aspergillus niger* in the cells of *Trichoderma reesei*. Bioresour Technol 102: 4568–4572. 10.1016/j.biortech.2010.12.099 21256746

[3] Rahman Z, Shida Y, Furukawa T, Suzuki Y, Okada H, Ogasawara W, et al (2009) Application of *Trichoderma reesei* cellulase and xylanase promoters through homologous recombination for enhanced production of extracellular β-Glucosidase I. Biosci Biotechnol Biochem 73: 1083–1089. 19420722 10.1271/bbb.80852

[4] Barnett CC, Berka RM, Fowler T (1991) Cloning and amplification of the gene encoding an extracellular β-glucosidase from *Trichoderma reesei*: evidence for improved rates of saccharification of cellulosic substrates. Biotechnol (NY) 9: 562–567. 1367522 10.1038/nbt0691-562

[5] Nakazawa H, Kawai T, Ida N, Shida Y, Kobayashi Y, Okada H, et al (2011) Construction of a recombinant *Trichoderma reesei* strain expressing *Aspergillus aculeatus* β-glucosidase 1 for efficient biomass conversion. Biotechnol Bioeng 109: 92–99. 10.1002/bit.23296 21830204

[6] Treebupachatsakul T, Nakazawa H, Shinbo H, Fujikawa H, Nagaiwa A, Ochiai N, et al (2015) Heterologously expressed *Aspergillus aculeatus* β-glucosidase in *Saccharomyces cerevisiae* is a cost-effective alternative to commercial supplementation of β-glucosidase in industrial ethanol production using *Trichoderma reesei* cellulases. J Biosci Bioeng. 10.1016/j.jbiosc.2015.05.002 26073313

[7] Treebupachatsakul T, Shioya K, Nakazawa H, Kawaguchi T, Morikawa Y, Shida Y, et al (2015) Utilization of recombinant Trichoderma reesei expressing Aspergillus aculeatus β-glucosidase I (JN11) for a more economical production of ethanol from lignocellulosic biomass. J Biosci Biotechnol. 10.1016/j.jbiosc.2015.04.015 26026380

[8] Teugjas H, Väljamäe P (2013) Selecting β-glucosidases to support cellulases in cellulose saccharification. Biotechnol Biofuels 6: 105 10.1186/1754-6834-6-105 23883540 PMC3726394

[9] Kubicek CP (1987) Improvement of a conidial endoglucanase and a plasmama-membrane-bound β-glucosidase in the induction of endoglucanase synthesis by cellulose in *Trichoderma reesei*. Gen Microbiol 133:1481–1487. 10.1099/00221287-133-6-1481 3117961

[10] Jeng WY, Wang NC, Lin MH, Lin CT, Liaw YC, Chang WJ, et al (2011) Structural and functional analysis of three β-glucosidases from bacterium *Clostridium cellulovorans*, fungus *Trichoderma reesei* and termite *Neotermes koshunensis*. J Struct Biol 173: 46–56. 10.1016/j.jsb.2010.07.008 20682343

[11] de Giuseppe PO, Souza Tde A, Souza FH, Zanphorlin LM, Machado CB, Ward RJ, et al (2014) Structural basis for glucose tolerance in GH1 β-glucosidases. Acta Crystallog D Biol Crystallogr 70: 1631–1639. 10.1107/S1399004714006920 24914974

[12] Saha BC, Bothast RJ (1996) Production, purification, and characterization of a highly glucose-tolerant novel β-glucosidase from *Candida peltata*. Appl Environ Microbiol 62: 3165–3170. 8795205 10.1128/aem.62.9.3165-3170.1996 PMC168111

[13] Zanoelo FF, Polizeli Mde L, Terenzi HF, Jorge JA (2004) Beta-glucosidase activity from the thermophilic fungus *Scytalidium thermophilum* is stimulated by glucose and xylose. FEMS Microbiol Lett 240: 137–143. 15522500 10.1016/j.femsle.2004.09.021

[14] Riou C, Salmon JM, Vallier MJ, Gunata Z, Barre P (1998) Purification, characterization, and substrate specificity of a novel highly glucose-tolerant β-glucosidase from *Aspergillus oryzae*. Appl Environ Microbiol 64: 3607–3614. 9758774 10.1128/aem.64.10.3607-3614.1998 PMC106471

[15] Fang Z (2010) Cloning and characterization of a β-glucosidase from marine microbial metagenome with excellent glucose tolerance. J Microbiol Biotechnol 20: 1351–1358. 20890102 10.4014/jmb.1003.03011

[16] Nascimento CV, Souza FH, Masui DC, Leone FA, Peralta RM, Jorge JA, et al (2010) Purification and biochemical properties of a glucose-stimulated β-D-glucosidase produced by *Humicola grisea* var. *thermoidea* grown on sugarcane bagasse. J Microbiol 48: 53–62. 10.1007/s12275-009-0159-x 20221730

[17] Souza FHM, Nascimento CV, Rosa JC, Masui DC, Leone FA, Jorge JA, et al (2010) Purification and biochemical characterization of a mycelial glucose- and xylose-stimulated β-glucosidase from the thermophilic fungus *Humicola insolens*. Process Biochem 45: 272–278.

[18] Uchima CA, Tokuda G, Watanabe H, Kitamoto K, Arioka M (2011) Heterologous expression and characterization of a glucose-stimulated β-glucosidase from the termite *Neotermes koshunensis* in *Aspergillus oryzae*. Appl Microbiol Biotechnol 89: 1761–1771. 10.1007/s00253-010-2963-y 21057947

[19] Lu J, Du L, Wei Y, Hu Y, Huang R (2013) Expression and characterization of a novel highly glucose-tolerant β-glucosidase from a soil metagenome. Acta Biochim Biophys Sin (Shanghai) 45: 664–673. 10.1093/abbs/gmt061 23752618

[20] Uchiyama T, Miyazaki K, Yaoi K (2013) Characterization of a novel β-glucosidase from a compost microbial metagenome with strong transglycosylation activity. J Biol Chem 288: 18325–18334. 10.1074/jbc.M113.471342 23661705 PMC3689974

[21] Ramani G, Meera B, Vanitha C, Rajendhran J, Gunasekaran P (2015) Molecular cloning and expression of thermostable glucose-tolerant β-glucosidase of *Penicillium funiculosum* NCL1 in *Pichia pastoris* and its characterization. J Ind Microbiol Biotechnol 42: 553–565. 10.1007/s10295-014-1549-6 25626525

[22] Yang F, Yang X, Li Z, Du C, Wang J, Li S (2015) Overexpression and characterization of a glucose-tolerant β-glucosidase from *T*. *aotearoense* with high specific activity for cellobiose. Appl Microbiol Biotechnol. 10.1007/s00253-015-6619-9 25957152

[23] Uchiyama T, Yaoi K, Miyazaki K (2015) Glucose-tolerant β-glucosidase retrieved from a Kusaya gravy metagenome. Front Microbiol 6: 548 10.3389/fmicb.2015.00548 26136726 PMC4468940

[24] Souza FHM, Inocentes RF, Ward RJ, Jorge JA, Furriel RP (2013) Glucose and xylose stimulation of a β-glucosidase from the thermophilic fungus *Humicola insolens*: A kinetic and biophysical study. J Mol Catal B-Enzym 94: 119–128.

[25] Lee HL, Chang CK, Jeng WY, Wang AH, Liang PH (2012) Mutations in the substrate entrance region of β-glucosidase from *Trichoderma reesei* improve enzyme activity and thermostability. Protein Eng Des Sel 25: 733–740. 10.1093/protein/gzs073 23077275

[26] Lowry OH, Rosebrough NJ, Farr AL, Randall RJ (1951) Protein measurement with the Folin phenol reagent. J Biol Chem 193: 265–275. 14907713

[27] Dixon M (1953) The determination of enzyme inhibitor constants. Biochem J 55: 170–171. 13093635 10.1042/bj0550170 PMC1269152

[28] Lineweaver H, Burk D (1934) The determination of enzyme dissociation constants. J Am Chem Soci 56: 658–666.

[29] Pei J, Pang Q, Zhao L, Fan S, Shi H (2012) *Thermoanaerobacterium thermosaccharolyticum* β-glucosidase: a glucose-tolerant enzyme with high specific activity for cellobiose. Biotechnol Biofuels 5: 1–10. 10.1186/1754-6834-5-31 22571470 PMC3395577

[30] Mai Z, Yang J, Tian X, Li J, Zhang S (2013) Gene cloning and characterization of a novel salt-tolerant and glucose-enhanced β-glucosidase from a marine streptomycete. Appl Biochem Biotechnol 169: 1512–1522. 10.1007/s12010-012-0080-3 23319184

[31] Michaelis L, Menten ML (1913) Die Kinetik der Invertinwirkung. Biochemische Zeitschrift 49: 333–369.

[32] Mandels M, Parrish FW, Reese ET (1962) Sophorose as an inducer of cellulase in *Trichoderma viride*. J Bacteriol 83: 400–408. 14469205 10.1128/jb.83.2.400-408.1962 PMC277742

[33] Saloheimo M, Kuja-Panula J, Ylosmaki E, Ward M, Penttila M (2002) Enzymatic properties and intracellular localization of the novel Trichoderma reesei β-glucosidase BGLII (Cel1A). Appl Environ Microbiol 68: 4546–4553. 12200312 10.1128/AEM.68.9.4546-4553.2002 PMC124102

