# Supplementary material for: Improvements in Glucose Sensitivity and Stability of Trichoderma reesei β-Glucosidase Using Site-Directed Mutagenesis
Source: PLoS One. 2016 Jan 20;11(1):e0147301. doi: 10.1371/journal.pone.0147301 (PMC4720395; doi:10.1371/journal.pone.0147301)
Supplement: S1 Fig — The recombinant enzymes (5 μg each) were subjected to electrophoresis. M, molecular mass markers. (PDF) [file pone.0147301.s001.pdf]

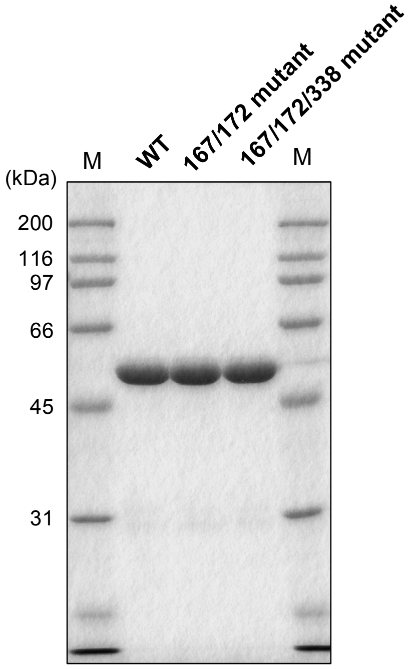

S1 Fig. SDS-PAGE analysis of the purified WT and the 167/172 and 167/172/338 mutants. The recombinant enzymes (5 µg each) were subjected to electrophoresis. M, molecular mass markers.
