# Supplementary material for: Improvements in Glucose Sensitivity and Stability of Trichoderma reesei β-Glucosidase Using Site-Directed Mutagenesis
Source: PLoS One. 2016 Jan 20;11(1):e0147301. doi: 10.1371/journal.pone.0147301 (PMC4720395; doi:10.1371/journal.pone.0147301)
Supplement: S2 Fig — Melting temperature was determined using Protein Thermal Shift Dye Kit (Thermo Fischer Scientific Inc., MA, USA) according to the instruction manual. A melting curve was created to trace the fluorescence (Ex/Em = 580 nm/610 nm) from 40 to 70°C at an increasing rate of 0.05°C per second. The melting temperature (Tm) was then obtained from a differential curve calculated from the melting curve. (PDF) [file pone.0147301.s002.pdf]

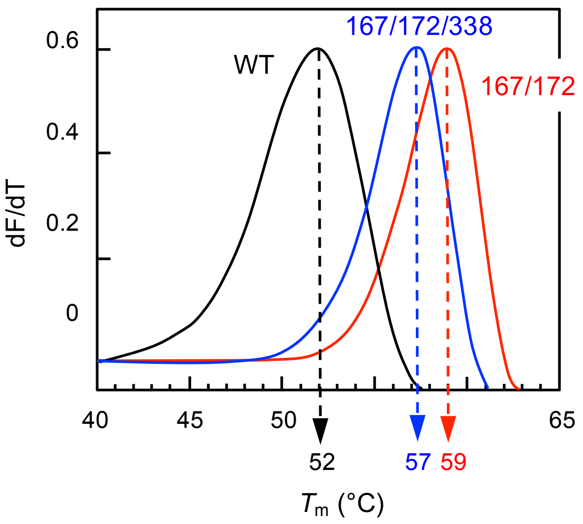

S2 Fig. Melting temperatures of WT and the 167/172 mutant.

Melting temperature was determined using Protein Thermal Shift Dye Kit (Thermo Fischer Scientific Inc., MA, USA) according to the instruction manual. A melting curve was created to trace the fluorescence (Ex/Em = 580 nm/610 nm) from 40 to 70°C at an increasing rate of 0.05°C per second. The melting temperature (T_m) was then obtained from a differential curve calculated from the melting curve.
