# Supplementary material for: Improvements in Glucose Sensitivity and Stability of Trichoderma reesei β-Glucosidase Using Site-Directed Mutagenesis
Source: PLoS One. 2016 Jan 20;11(1):e0147301. doi: 10.1371/journal.pone.0147301 (PMC4720395; doi:10.1371/journal.pone.0147301)
Supplement: S4 Fig — (A) WT, (B) The 167/172 mutant. The same data from Fig 4 were plotted. Plots are averages of independent triplicates. (PDF) [file pone.0147301.s004.pdf]

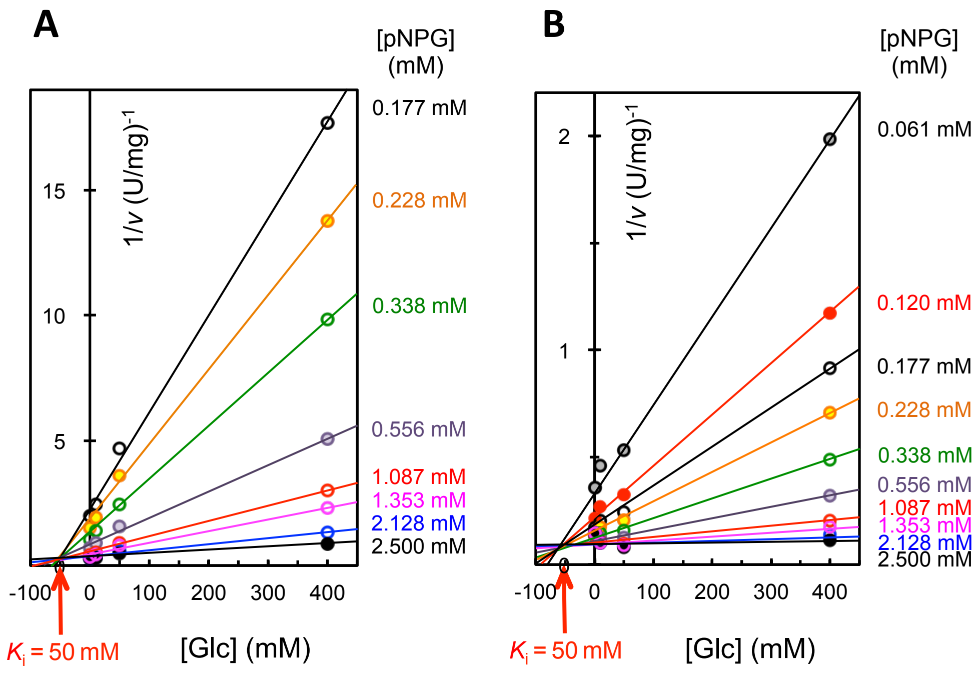

S4 Fig. Dixon plot analysis for determination of parameters in glucose inhibition.

(A) WT, **(B)** The 167/172 mutant. The same data from Fig 4 were plotted. Plots are averages of independent triplicates.
